# Interaction of bacterial fatty-acid-displaced regulators with DNA is interrupted by tyrosine phosphorylation in the helix-turn-helix domain

**DOI:** 10.1093/nar/gkt709

**Published:** 2013-08-10

**Authors:** Abderahmane Derouiche, Vladimir Bidnenko, Rosa Grenha, Nathalie Pigonneau, Magali Ventroux, Mirita Franz-Wachtel, Sylvie Nessler, Marie-Françoise Noirot-Gros, Ivan Mijakovic

**Affiliations:** ^1^INRA, UMR1319 Micalis, 78350 Jouy-en-Josas, France, ^2^Institut de Biochimie et Biophysique Moléculaire et Cellulaire, Université Paris-Sud 11, 91405 Orsay, France, ^3^Proteome Center Tübingen, University of Tübingen, 72076 Tübingen, Germany and ^4^Department of Chemical and Biological Engineering, Chalmers University of Technology, 41296 Gothenburg, Sweden

## Abstract

Bacteria possess transcription regulators (of the TetR family) specifically dedicated to repressing genes for cytochrome P450, involved in oxidation of polyunsaturated fatty acids. Interaction of these repressors with operator sequences is disrupted in the presence of fatty acids, and they are therefore known as fatty-acid-displaced regulators. Here, we describe a novel mechanism of inactivating the interaction of these proteins with DNA, illustrated by the example of *Bacillus subtilis* regulator FatR. FatR was found to interact in a two-hybrid assay with TkmA, an activator of the protein-tyrosine kinase PtkA. We show that FatR is phosphorylated specifically at the residue tyrosine 45 in its helix-turn-helix domain by the kinase PtkA. Structural modelling reveals that the hydroxyl group of tyrosine 45 interacts with DNA, and we show that this phosphorylation reduces FatR DNA binding capacity. Point mutants mimicking phosphorylation of FatR *in vivo* lead to a strong derepression of the *fatR* operon, indicating that this regulatory mechanism works independently of derepression by polyunsaturated fatty acids. Tyrosine 45 is a highly conserved residue, and PtkA from *B. subtilis* can phosphorylate FatR homologues from other bacteria. This indicates that phosphorylation of tyrosine 45 may be a general mechanism of switching off bacterial fatty-acid-displaced regulators.

## INTRODUCTION

Bacterial protein-tyrosine kinases (BY-kinases) are ubiquitous bacterial enzymes that phosphorylate a number of cellular protein substrates and interact with exopolysaccharide synthesis and export machineries (1,2). In Proteobacteria, BY-kinase genes encode large transmembrane proteins, with an extracellular domain of variable size, and an intracellular C-terminal catalytic domain responsible for the kinase activity (1,3). In Firmicutes, the transmembrane domain and the kinase domain are encoded by separate genes, but interaction with the transmembrane protein is necessary for the activity of the kinase domain (1,3). Recently, X-ray crystal structures of BY-kinases have elucidated the mechanism of this activation, based on the stabilization of the adenosine triphosphate (ATP)-binding site by the cytosolic domain of the transmembrane protein (4). In their capacity of exopolysaccharide co-polymerases, BY-kinase proteins play a key role in virulence of bacteria. For example, autophosphorylation of the BY-kinase CpsD was shown to promote attachment of capsular polysaccharide to the cell wall, which is required for invasive properties of *Streptococcus pneumoniae* (5). In terms of substrate phosphorylation, arguably the most extensively characterized BY-kinase from Firmicutes is the *B. subtilis* kinase PtkA (6). PtkA is a promiscuous kinase, as it has been shown to phosphorylate and thereby regulate the activity of a number of target proteins. PtkA-dependent phosphorylation activates the uridine-diphosphate (UDP) glucose dehydrogenase Ugd (7,8) and increases the DNA binding affinity of the single-stranded DNA-binding protein SsbA (9). PtkA also influences localization of a number of other protein substrates (10) and is potentially involved in biofilm formation (11) and regulation of the cell cycle (12). The transmembrane activator of PtkA, necessary for substrate phosphorylation, is designated TkmA (6). To identify potential new substrates and/or interaction partners of PtkA and TkmA, we have performed a yeast two-hybrid assay. Interestingly, among other identified interactants, TkmA interacted very specifically with FatR, a protein involved in regulating the metabolism of polyunsaturated fatty acids.

Polyunsaturated fatty acids are structural components of the cell membrane, but in high concentrations, they can be toxic for bacteria. A specific adaptive response of soil-dwelling bacteria to toxic fatty acids issued from plants was first described in *Bacillus megaterium* (13). This bacterium possesses a cytochrome P450 fatty acid monooxygenase, designated CYP102, which can detoxify the fatty acids by hydroxylation (14). The expression of the gene encoding CYP102 is regulated by a transcriptional repressor Bm3R1 (TetR family of regulators), which prevents the transcription by roadblock when bound to the operator site (15). In the presence of polyunsaturated fatty acids, Bm3R1 is displaced from the operator, and expression of the gene *cyp102* is induced (13). The orthologue of *B. megaterium* Bm3R1 in *B. subtilis* is FatR (synonyms BscR, YrhI) (16). The CYP102 orthologue in *B. subtilis* is Cyp102A3 (synonym YrhJ, CypE) (16). *B. subtilis* Cyp102A3 has been enzymatically characterized and found to hydroxylate long-chain unsaturated and branched-chain fatty acids in subterminal positions (17,18). However, in *B. subtilis*, Cyp102A3 is not essential for detoxification of fatty acids, and it has been speculated that it may have other metabolic roles (16). The regulator FatR of *B. subtilis* represses the operon *fatR-cyp102A3* by binding to an 18-bp palindrome sequence between the transcription initiation site and the Shine–Dalgarno sequence, and can also be displaced from the target sequence in the presence of fatty acids (19). Fatty acids are, however, not the only regulatory input for the *fatR-cyp102A3* operon in *B. subtilis*, as it is also under the control of the AbrB transcription regulator, and can be influenced by a presently unidentified diffusible substance (20). Because FatR was found to interact with the BY-kinase activator TkmA, we explored the possibility that it could also be regulated by tyrosine phosphorylation.

In this report, we demonstrate that FatR is phosphorylated by the kinase PtkA. Phosphorylation takes place at the FatR residue tyrosine 45, and disrupts the interaction of the protein with DNA. Accordingly, the *in vivo* consequence of FatR phosphorylation is derepression of the *fatR-cyp102A3* operon. Because tyrosine 45 is highly conserved, PtkA was able to phosphorylate FatR orthologues from other *Bacilli.* We, therefore, propose that BY-kinase-dependent phosphorylation of tyrosine 45 might represent a general mechanism for inactivation of fatty-acid-displaced regulators in bacteria.

## MATERIALS AND METHODS

### Bacterial strains and growth conditions

*Escherichia coli* NM522 was used for gene cloning. The chaperone overproducing strain *E. coli* M15 carrying pREP4-GroESL (21) was used for overproduction of proteins. *B. subtilis* 514 (*B. subtilis 168 trp^+^ P_r_::Neo^R^*) was used for *in vivo* mutant construction. *E. coli* and *B. subtilis* strains were grown in Luria–Bertani (LB) medium with shaking at 37°C. Ampicillin (100 µg/ml) and kanamycin (25 µg/ml) for *E. coli*, and erythromycin (1 µg/ml), neomycin (5 µg/ml) and phleomycin (2 µg/ml) for *B. subtilis* were added when appropriate.

### DNA manipulations and strain construction

Genes were polymerase chain reaction (PCR)-amplified from genomic DNA using specific primers with restriction sites (Supplementary Table S1). These included *fatR* and *yhgD* from *B. subtilis*, *fatR* homologues from *Bacillus thuringiensis*, *Bacillus cereus*, *B. megaterium* and *Lactobacillus casei*, as well as the *ptkA* homologue from *B. megaterium* (*bmQ-1130*). The point-mutations *fatR* Y45F and *fatR* Y45E were obtained using two partially overlapping mutagenic primers. For heterologous expression, PCR products were inserted between the BamHI and PstI sites of the vector pQE-30 (Qiagen), introduced *in E. coli* NM522 and verified by sequencing. Construction of the *fatR* Y45F and *fatR* Y45E mutants at the natural *fatR* locus was performed using the modified mutation delivery method of Fabret *et al.* (22,23). Briefly, the DNA fragments were amplified from the *B. subtilis 168* chromosome using the pairs of oligonucleotides *fatRfwd–fatRY45F-R* and *fatRY45F-F–fatRrev* for the Y45F mutation and *fatRfwd–fatRY45E-R* and fatRY45E*-F–fatRrev* for the Y45E mutation. Joining PCR, performed with primers *fatRfwd* and *fatRrev*, was used to link these fragments to the extremities of the insertion cassette, which contained the phleomycin (Phleo) resistance marker and the phage lambda *cI* repressor gene. The resulting PCR product was used to transform the competent BS514 (*B. subtilis 168 trp^+^ P_r_::Neo^R^*) cells containing the neomycin (Neo) resistance gene under control of the lambda P_r_ promoter. The transformants were first selected for the phleo-resistance and the neo-sensitivity, and finally the counter-selection for neo-resistance and phleo-sensitivity was applied to select clones that had lost the insertion cassette via recombination between the flanking direct repeats. The deletion of *fatR* gene was performed similarly using the pairs of oligonucleotides *yrhH* forward–Del*-fatR-R* and Del*-fatR-F–yrhJ* reverse (Supplementary Table S1)*.* The partially complementary primers Del*-fatR-F* and Del*-fatR-R* were designed to introduce an in-frame deletion between codons 35 and 162 of the *fatR* gene. Thus, Δ*fatR* mutant encodes a short peptide composed of the first 34 and the last 32 amino acids of FatR*.* The obtained *fatR* mutants were checked by sequencing. For *lacZ* fusion with *cyp102A3* gene, a 0.5-kb fragment corresponding to the central region of *cyp102A3* was PCR-amplified from genomic DNA and inserted between the EcoRI and BamHI sites of pMUTIN2. *B. subtilis wild type, ΔptkA* (12), *fatR* Y45E *and fatR* Y45E were transformed with the pMUTIN2 construct and selected for Erm resistance. BSB1 strain, which is a tryptophan-prototrophic (Trp+) derivative of the 168 trpC2 strain (24), expressing a functional sequential peptide affinity (SPA) tagged FatR protein was constructed as follows. A 400-bp DNA fragment at the 3′ end of the *fatR* gene was cloned in the pMUTIN-SPA plasmid (25). The resulting plasmid was integrated into the *fatR* locus, to generate a strain expressing FatR-SPA as the sole source of FatR. The expression of the FatR-SPA protein was checked by Western blot using an anti-FLAG antibody. The same procedure was followed in the Δ*ptkA* strain (12). All *B. subtilis* strains used in this study are listed in Supplementary Table S2.

### Yeast two-hybrid

The yeast two-hybrid phenotypic assay for TkmA and PtkA interactants was performed as described previously (26). Briefly, fusion of TkmA or PtkA with the DNA-binding domain of Gal4 was used to screen the *B. subtilis* yeast two-hybrid library (27). The screen was performed in yeast haploid cells, and interacting phenotypes were screened for the ability of mated diploid cells to grow on the selective medium (SD-LUH).

### Synthesis and purification of tagged proteins

All proteins were synthesized as 6xHis N-terminal fusions in *E. coli* M15 harbouring pREP4-GroESL, except for 6xHis-FatR wild type, which was overproduced directly in *E. coli* NM522. Cultures were grown with shaking at 37°C to OD_600_ of 0.5, expression was induced with 1-mM isopropyl β-d-1-thiogalactopyranoside and cells were grown for an additional 3 h. 6xHis-tagged proteins were purified on Ni-NTA columns (Qiagen) as described previously (7). Protein aliquots were stored at −80°C in a buffer containing 50 mM Tris-HCl, pH 7.5, 100 mM NaCl and 10% glycerol.

### *In vitro* phosphorylation assays

Phosphorylation assays were performed essentially as described previously for substrates of *B. subtilis,* PtkA and TkmA (7). A typical 40 µl reaction contained 1 µM PtkA, 1 µM TkmA-NCter and 5 µM FatR (or FatR Y45E/F), 50 µM [γ-^32^P] ATP (20 µCi/mmol), 1 mM MgCl_2_ and 100 mM Tris-HCl, pH 7.5. The same reaction was repeated with PtkA and FatR homologues from *B. megaterium*. The conditions described above were also used to perform the phosphorylation assays of *FatR* homologues from *B. thuringiensis*, *B. cereus*, *B. megaterium* and *L. casei*, as well as the *B*. *subtilis* protein YhgD. Reactions were incubated at 37°C for 60 min and stopped by adding sodium dodecyl sulphate-polyacrylamide gel electrophoresis loading buffer and heating at 100°C for 5 min. Proteins were separated by electrophoresis on sodium dodecyl sulphate-polyacrylamide gel electrophoresis (12% polyacrylamide), then washed by boiling in 0.5 M HCl for 10 min to reduce the background and then dried. Signals were visualized with the FUJI PhosphoImager.

### Mass spectrometry analysis of phosphorylation sites

Hundred micrograms of 6xHis-FatR wild-type protein was phosphorylated *in vitro* using the protocol described above (without radioactive ATP). Purified FatR was digested in solution with trypsin as described elsewhere (28). Ten percent of the peptide mixture was desalted using C18 StageTips (29) and analysed directly by LC-MS/MS, whereas 90% was subjected to phosphopeptide enrichment by titanium dioxide chromatography as described previously (30), with the following modifications: phosphopeptide elution from the beads was performed three times with 100 ml 40% ammonia hydroxide solution in 60% acetonitrile at a pH of >10.5. Analysis of peptides and phosphopeptides was done on a Proxeon Easy-LC system (Proxeon Biosystems, Denmark) coupled to an LTQ-Orbitrap-XL (Thermo Fisher Scientific, Germany) equipped with a nanoelectrospray ion source (Proxeon Biosystems, Denmark) as described previously (31). The five most intense precursor ions were fragmented by activation of neutral loss ions at −98, −49 and −32.6 relative to the precursor ion (multistage activation). Mass spectra were analysed using the software suite MaxQuant, version 1.0.14.3 (32). The data were searched against a target-decoy *E. coli* database including the 6xHis- FatR sequence and 262 frequently observed contaminants. Besides acetylation at the N-terminus and oxidation of methionine, phosphorylation of serine, threonine and tyrosine were set as variable modifications. Carbamidomethylation of cysteine was set as fixed modification. Initial precursor mass tolerance was set to 7 ppm at the precursor ion and 0.5 Da at the fragment ion level. False discovery rates were set to 1% at peptide, phosphorylation site and protein group level. Phosphorylation events detected within a given peptide with a localization probability of at least 0.75 were considered to be assigned to a specific amino acid residue.

### Circular dichroism measurements

All circular dichroism (CD) experiments were performed on a Jasco J-810 spectropolarimeter (Jasco) at 20°C in a 0.1-mm path length cuvette (106-QS, Hellma Analytics). Proteins purified in 50 mM Tris–HCl, pH 7.5, 100 mM NaCl and 10% glycerol were dialyzed against 50 mM ammonium bicarbonate and lyophilized. The lyophilized samples were resuspended in 25 mM HEPES, pH 7.5, 100 mM NaCl at a concentration of 2.4 mg/ml for FatR-wt, 1.3 mg/ml for FatR(Y45E) and 1.5 mg/ml for FatR(Y45F). The samples were diluted in water to a final concentration of ≈1.25 mg/ml (≈55 µM) for the measurements. The cuvette-holding chamber was under a constant stream of N_2_ gas flow to avoid water condensation from forming on the cuvette exterior. The CD spectra shown for each protein correspond to the average of five continuous scans from 185 to 260 nm, collected at 20 nm/min. The data were processed using Spectra Analysis from the Jasco Spectra ManagerTM Software.

### DNA-binding assay

Electrophoretic mobility-shift assays were done with 24-bp double-stranded DNA probe containing the putative *fatR* operator (16). It was constructed by annealing two synthetic oligonucleotides BsOP1 and BsOP2 (Supplementary Table S1). Different ratios (indicated in the figure legend) of the oligonucleotide and FatR wild type, FatRY45F and FatRY45E were mixed in 100 mM Tris–HCl, pH 7.5, incubated for 15 min at 37°C and loaded on 8% polyacrylamide gel without SDS. After migration in a Tris-glycine buffer (2.5 mM Tris and 192 mM glycine), the gel was incubated for 45 min with shaking in 100 ml of Tris-glycine buffer with 1 µg/ml of ethidium bromide to visualize DNA.

### β-Galactosidase assay

Hundred milliliters of LB was inoculated with overnight *B. subtilis* culture to OD_600_ of 0.02, and grown with shaking at 37°C. To induce the *fatR-cyp102A3* operon, linoleic acid in dimethyl sulfoxide (DMSO) and NaCl were added to the cultures with a final concentration of linoleic acid 10 μM, DMSO 0.1% (v/v) (16) and NaCl 0.4 M. DMSO was also added to the control culture. At time points indicated in the Figure 4B, 2-ml of samples was taken, spun down (10 000g, 2 min) and cell pellets were stored at −20°C. The pellet was resuspended in 0.5-ml Z-buffer (60 mM Na_2_HPO_4_.7H_2_0, 0.04 mM NaH_2_PO_4_.H20, 10 mM KCl, 1 mM MgSO_4_ and 50 mM β-mercaptoethanol, pH 7.0). 0.5 ml cell suspension was treated with 30 µg lysozyme and 3 µg DNase for 15 min at 37°C. Reaction was started by addition of 0.2 ml of 4 mg/ml ortho-nitrophenyl-β-galactoside (ONPG) and stopped with 0.5 ml of 1 M Na_2_CO_3_. Miller units were calculated as described (33).

### ChIP-on-chip analysis

Cells expressing the FatR-SPA protein were grown in 2 L of LB medium (erythromycin, 0.6 mg/L; IPTG, 0.5 mM) with vigorous shaking at 37°C. At OD_600_ of 0.9, the cells were treated with formaldehyde 0.6% final concentration, and cross-linking was carried out for 20 min at room temperature before quenching with 125 mM glycine (final concentration). Two biological replicates were analysed for each strain [wild type (WT) and Δ*ptkA*]. The ChIP-chip was performed using a procedure described previously (34). Data processing was performed as previously described (35). Signal intensities were expressed as log2 ratio (ChIP-DNA/Control-DNA) and corrected for dye bias using Loess regression on the MA plot (36).

### Structural examination of FatR homologues

BLAST-PDB was used to detect FatR homologues among the resolved protein structures in the PDB database. Three best hits, 3HE0, 2GEN and 1JT0, were selected for further analysis. All structural figures were created with CCP4MG (37), and superposition was done using the CCP4 suite (38).

## RESULTS

### FatR is phosphorylated by the BY-kinase PtkA at the residue tyrosine 45

**Figure 1.**
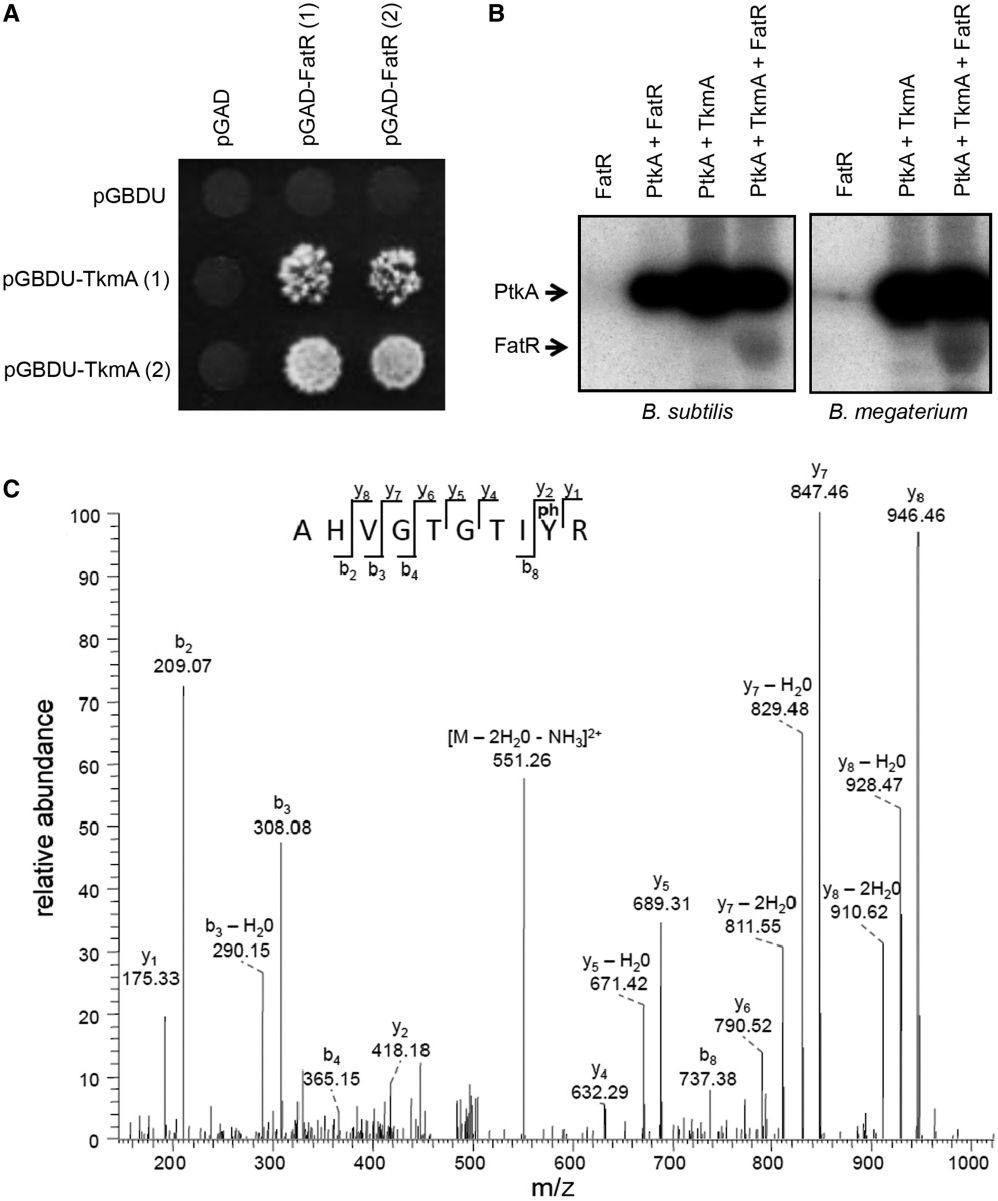
PtkA phosphorylates FatR at the residue tyrosine 45. (**A**) Specific interaction of the C-terminal part of FatR (residues 101–194) with TkmA in the yeast two-hybrid assay. Gene *fatR* 101–194 was cloned in the plasmids pGAD as a translational fusion with activating domain of the *gal4* regulator, and *tkmA* was cloned in pGBDU, in fusion with the binding domain of *gal4.* Two clones for each construct were tested, designated (1) and (2). Vectors without *fatR* 101–194 and *tkmA* were used as negative controls. Eight days after the drop of yeast cells on the selective (−)LUH SD medium, the development of colonies was observed for tested strains expressing interacting proteins. (**B**) *In vitro* phosphorylation assay of FatR. Presence of purified proteins is indicated above each reaction lane. All reactions contained [γ-^32^P] ATP, MgCl_2_ and Tris-HCl, pH 7.5: concentrations and reaction conditions are given in Materials and Methods section. In the left, PtkA and FatR are from *B. subtilis*, and in the right, they are from *B. megaterium*. TkmA is from *B. subtilis* in all reactions. (**C**) After *in vitro* phosphorylation of *B. subtilis* FatR by PtkA, the sample was digested in solution with trypsine, and phosphopeptides were enriched by titanium dioxide chromatography and subjected to mass spectrometry. The spectrum shows the fragmentation pattern of the FatR phosphopeptide AHVGTGTIY(ph)R phosphorylated at the tyrosine 45.

To search for new interactants of PtkA and TkmA, we performed a two-hybrid screen against the *B. subtilis* genomic library (27). Among others (data not shown), we detected a specific interaction between TkmA and the C-terminal domain (residues 101–194) of the fatty-acid-displaced regulator FatR. The results of specificity test for this interaction are presented in Figure 1A. Development of colonies was observed only for strains containing the plasmid pGAD, with the activating domain of the *gal4* gene fused to the *tkmA* and the pGBDU plasmid with the binding domain of the *gal4* fused to the C-terminal fragment of *fatR*. Negative controls with empty plasmids confirm that the simultaneous presence of FatR and TkmA was required for growth. We concluded that TkmA and FatR interact in the yeast two-hybrid assay, mimicking the *in vivo* conditions. Because TkmA is known to activate the BY-kinase PtkA and promote substrate phosphorylation presumably in a ternary complex (TkmA-PtkA-substrate) (7), we tested whether PtkA would be able to phosphorylate FatR in the presence of TkmA. For this, we set up an *in vitro* phosphorylation assay, using purified 6xHis-tagged proteins. Proteins were mixed with [γ-^32^P] ATP, and incorporation of radioactive phosphate was revealed by autoradiography. As can be observed in Figure 1B (left), FatR was not able to autophosphorylate in the presence of [γ-^32^P] ATP. In the absence of TkmA, PtkA could not phosphorylate FatR. However, a radioactive signal associated to FatR appeared when both PtkA and TkmA were present in the reaction, indicating that FatR could be phosphorylated by PtkA. Since the FatR homologue from *B. megaterium* (Bm3R1) was the first characterized member of this regulator family, we also examined whether it can be phosphorylated by its cognate kinase (product of the gene *bmQ-1130* identified by BLAST). We purified the *B. megaterium* FatR and PtkA proteins and performed the same type of *in vitro* phosphorylation assay. The result was identical to that obtained with *B. subtilis* proteins, and *B. megaterium* FatR was phosphorylated by the cognate PtkA in the presence of *B. subtilis* TkmA (Figure 1B, right). To determine the exact site of *B. subtilis* FatR phosphorylation, we performed the phosphorylation reaction in exactly the same conditions, but without radioactive ATP. We then attempted to detect the phosphorylation site on FatR using mass spectrometry analysis. Phosphorylation was unambiguously detected at the residue tyrosine 45, with the probability score of 0.976 (Figure 1C). We thus concluded that PtkA catalyzes a specific phosphorylation of FatR at the residue tyrosine 45.

### FatR tyrosine 45 is highly conserved and situated in the DNA-binding domain

**Figure 2.**
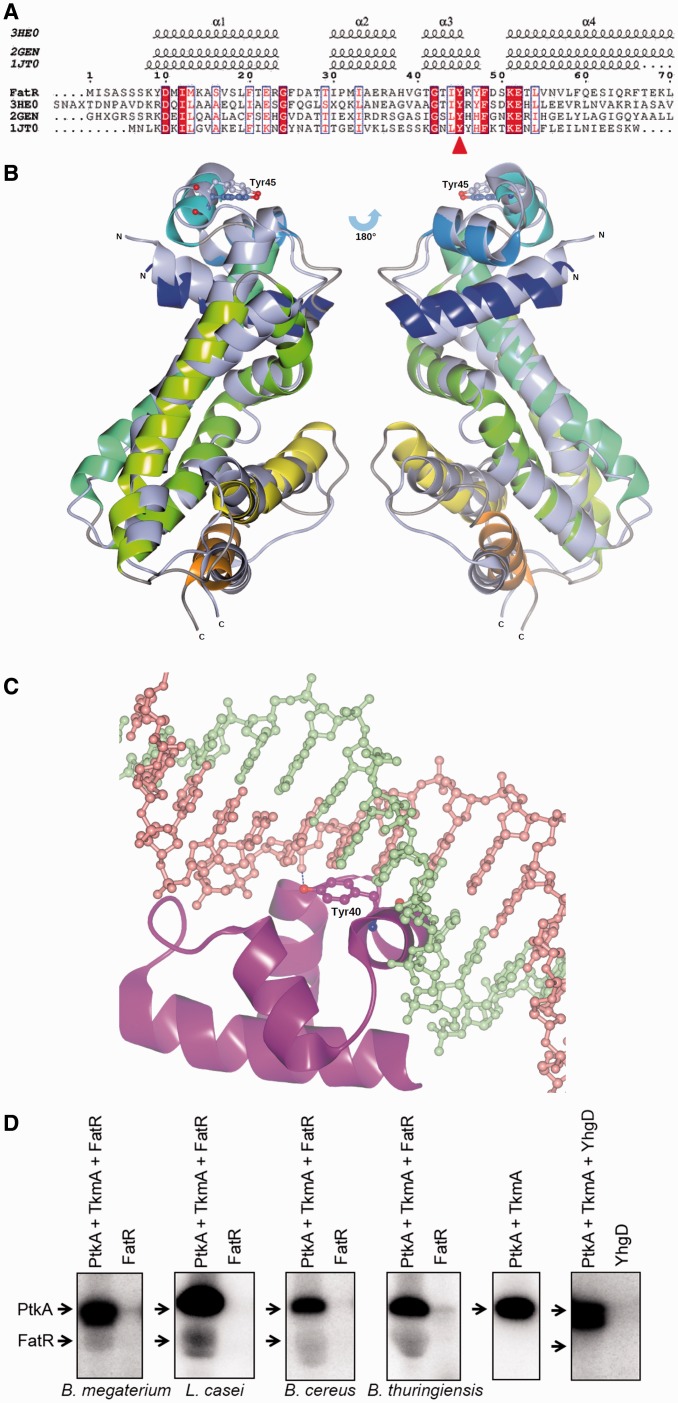
Residue tyrosine 45 of FatR is conserved and involved in interaction with DNA. (**A**) Alignment of the helix-turn-helix domain of FatR with its homologues from *Vibrio parahaemolyticus* (3HE0), *P. aeruginosa* (2GEN), and *S. aureus* (1JT0). Position of tyrosine 45 of FatR is indicated by a red arrow. (**B**) Ribbon diagrams of structures of 3HE0 and 2GEN superimposed. The position of the residue tyrosine 45 is highlighted as a ball and stick model. 2GEN is in rainbow from blue to red, and 3HE0 is in green. (**C**) Zoom-in view of the interaction of Tyr40 in 1JT0 (conserved residue Tyr45 in FatR) with its HTH-DNA-binding domain. Tyr45 is highlighted as a ball and stick model in magenta, DNA is presented in ball and stick model, green and coral. Hydrogen bond is in blue dashes. (**D**) *In vitro* phosphorylation assay of FatR homologues from different bacteria. Presence of purified proteins is indicated above each reaction lane, and the species of origin below the lanes (no origin is indicated for *B. subtilis* proteins). All reactions contained [γ-^32^P] ATP, MgCl_2_ and Tris–HCl, pH 7.5: concentrations and reaction conditions are given in Materials and Methods section.

There are a number of DNA-binding proteins with resolved 3D structures in the PDB database that exhibit extensive homology with the FatR helix-turn-helix domain. Based on the PDB-Blast, we identified three bacterial transcriptional regulators that are particularly close structural homologues: 3HE0 (transcriptional regulator from *Vibrio parahaemolyticus*, unpublished structure deposited in PDB database), 2GEN (transcriptional regulator from *Pseudomonas aeruginosa*, unpublished structure deposited in PDB database) and 1JT0 (*Staphylococcus aureus* multidrug-binding protein QacR) (39). An alignment comparison of FatR with the helix-turn-helix domains of these homologues shows that the phosphorylated residue tyrosine 45 is situated in the helix-turn-helix domain, and is highly conserved (Figure 2A). In Figure 2B, we show the structures of 3HE0 and 2GEN superimposed. The position of the residue tyrosine 45 is highlighted; this residue is located on the surface of the protein and accessible to solvent (and to the kinase). The hydroxyl group of the residue tyrosine 40 of the *S.aureus* QacR (aligning with residue tyrosine 45 of FatR) was found to form a hydrogen bond with the DNA (Figure 2C). Phosphorylation of the tyrosine 45 should thus be expected to mask the hydroxyl group, introduce a negative charge at the protein-DNA interaction surface and disrupt the interaction of FatR with DNA. Because the residue tyrosine 45 is highly conserved in the members of the FatR family, we decided to test whether PtkA could phosphorylate some FatR homologues from closely related bacteria. We purified FatR orthologues from *B. thuringiensis*, *B. cereus, B. megaterium* and *L. casei* and tested them in the *in vitro* phosphorylation assay with PtkA and TkmA from *B. subtilis*. All examined proteins tested positive for phosphorylation by PtkA (Figure 2D). To assess the specificity of the PtkA–FatR relationship, we attempted to use PtkA/TkmA to phosphorylate another member of the TetR/AcrR regulator family with a conserved Y45, YhgD from *B. subtilis.* This control yielded a negative result; YhgD was not recognized as a substrate of PtkA.

### Non-phosphorylated state of FatR tyrosine 45 is essential for DNA binding

**Figure 3.**
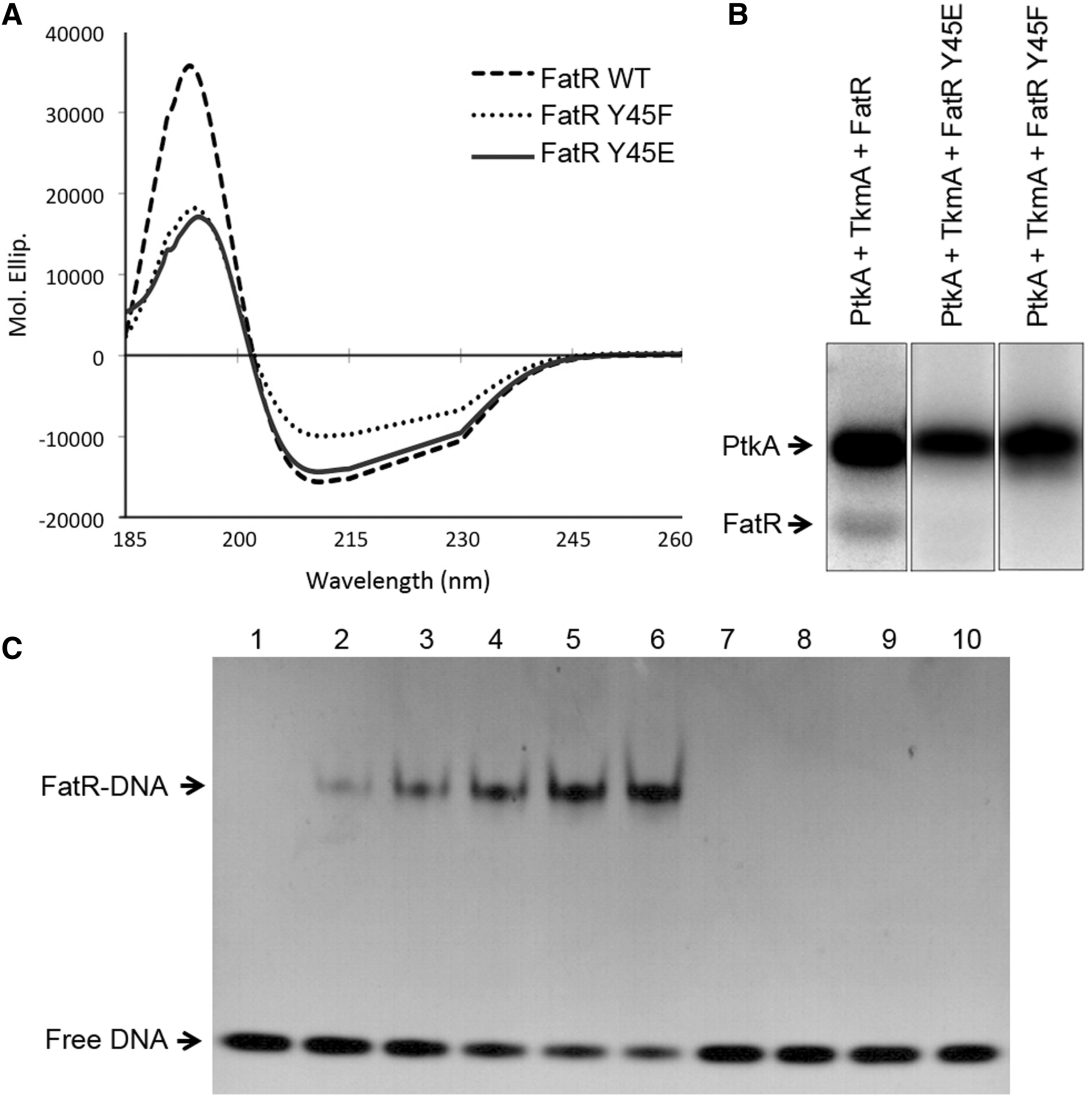
Hydroxyl group of FatR tyrosine 45 is essential for DNA binding. (**A**) CD spectra of FatR-WT (dashed line), FatR-Y45F (dotted line) and FatR-Y45E (solid line). Graphs are plotted in units of mean residue ellipticity (θ in 10^5 ^deg cm^2 ^mol^−1^) at the respective wavelength (nm). (**B**) *In vitro* phosphorylation assay of different versions of FatR. Presence of purified proteins is indicated above each reaction lane. All reactions contained [γ-^32^P] ATP, MgCl_2_ and Tris-HCl, pH 7.5: concentrations and reaction conditions are given in Materials and Methods section. (**C**) Binding of different versions of FatR to its operator sequence analysed by electrophoretic mobility-shift assays. All reactions contained 30 pmol of DNA. Lane 1 is a control with no protein. Wild-type FatR is present in lanes 2–6, at final concentration of 120, 240, 480, 960 and 1920 pmol, respectively. This corresponds to molar ratios of protein to DNA of 1:4, 1:8, 1:16, 1:32 and 1:64, respectively. Lanes 7 and 8 contained 960 and 1920 pmol of FatR Y45F (protein:DNA ratios of 1:32 and 1:64), respectively. Lanes 9 and 10 contained 960 and 1920 pmol of FatR Y45E (protein:DNA ratios of 1:32 and 1:64), respectively. The signals corresponding to free DNA and FatR-DNA complex are indicated by arrows.

To explore the hypothesis that phosphorylation could disrupt DNA binding of FatR, we replaced the residue tyrosine 45 with either phenylalanine or glutamate. Both these mutants do not possess the hydroxyl group crucial for interaction with DNA, and were thus expected to bind DNA less efficiently. The glutamate mutant bears a negative charge at neutral pH, which should further destabilize the interaction with the negatively charged DNA. To check whether these point mutants are properly folded, we performed a CD experiment. The CD spectra of both FatR-Y45E and FatR-Y45F mutant proteins were similar to that of the wild-type FatR (Figure 3A). The three protein samples displayed CD spectra, with a positive peak at about 190 nm and negative peaks at about 208 and 230 nm, characteristic of a mostly α-helical fold (40). Both available algorithms for analysis of CD spectra (41,42) calculated the same proportion of α-helices of ∼89% for all three forms of FatR. We concluded that mutating the tyrosine 45 did not induce any unfolding of FatR. This is in agreement with the structural model predicting that Y45 is exposed at the protein surface (Fig 2C). Next, we used the mutants FatR-Y45F and FatR-Y45E to confirm the mass spectrometry data, using the radioactive *in vitro* phosphorylation assay. Both these mutants lack the hydroxyl group of tyrosine 45, and are therefore expected to be non-phosphorylatable. In the *in vitro* assay, FatR-45F and FatR-Y45E lost >95% of the radioactive signal compared with the wild type (Figure 3B), indicating that tyrosine 45 is indeed the main phosphorylation site. To assign the remaining 5% of the radioactive signal, we performed an additional mass spectrometry analysis (as described in Materials and Methods section). We performed another cycle of phosphopeptide enrichment with TiO_2_ and reprocessed all data together. We thus detected three extremely low occupancy phosphorylation sites: Y99, Y112 and Y131 (respective localization probabilities: 0.902, 0.999 and 0.999). Finally, we examined the influence of FatR point mutations on its capacity to bind DNA. Because the hydroxyl group of tyrosine 45 is involved in a hydrogen bond with DNA (Figure 2C), both FatR-Y45F and FatR-Y45E should be unable to form this hydrogen bond. By consequence, their DNA binding was expected to be impaired. To test this, we used the standard electrophoretic mobility-shift assay with the FatR operator sequence (Figure 3C). Although increasing the molar ratio of wild-type FatR to DNA produced a clear shift, both FatR-Y45F and FatR-Y45E were unable to bind DNA, even at the protein:DNA molar ratio of 64:1. We thus concluded that the non-phosphorylated form of FatR, with the hydroxyl group of tyrosine 45 available for interaction, is the form that binds DNA preferentially.

### Phosphorylation of FatR tyrosine 45 leads to derepression of the *fatR-cyp102A3* operon *in vivo*

**Figure 4.**
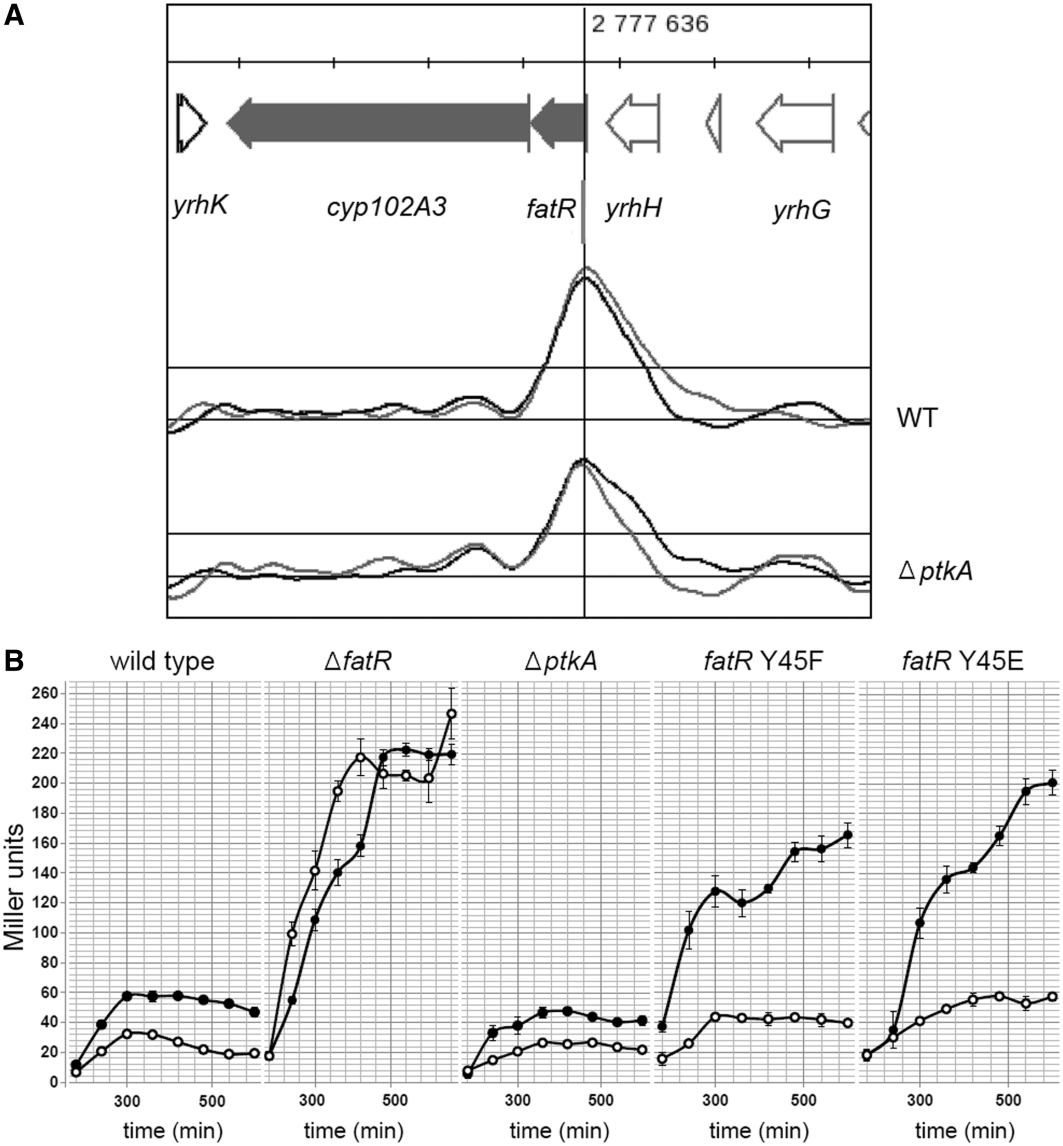
Phosphomimetic mutant *fatR* Y45E leads to a strong derepression of the *fatR-cyp102A3* operon *in vivo*. (**A**) The unique FatR binding site detected by ChIP-on-chip on the *B. subtilis* genome in the WT and the Δ*ptkA* strain. Genes in the *B. subtilis* genome are represented on top. The ChIP-on-chip profile of interaction with FatR-SPA is given for the WT and Δ*ptkA* strain (replicates 1 and 2 coloured in black and grey, respectively). The peaks correspond to enrichment of a specific DNA sequence co-purified in complex with FatR-SPA. The vertical line indicates the position of the binding site. (**B**) Promoter activity of the operon *fatR-cyp102A3* measured as Miller units of the β-galactosidase reporter gene. Experiments were performed using the following strains: WT, Δ*ptkA*, Δ*fatR*, *fatR* Y45E and *fatR* Y45F. Noninduced strains were grown in LB with DMSO 0.1% (open circles), and 10 µM linoleate (in DMSO 0.1%) and 0.4 M NaCl were added to induce the promoter (filled circles). The results represent the maximal induction reached in each strain, at the end of exponential phase. Average of three independent measurements is shown, and standard deviations are indicated with error bars.

Because the loss of the hydroxyl group of tyrosine 45 impairs DNA binding, we hypothesized that phosphorylation of FatR might constitute a signal to derepress its target operon(s). So far, the only reported target operon for FatR regulation is *fatR-cyp102A3* (16,17). To examine whether there are other targets for FatR, we first performed an exhaustive search for FatR binding sequences on the entire *B. subtilis* chromosome. To do this, we replaced the WT *fatR* gene with a copy encoding FatR-SPA. This protein was cross-linked to its chromosomal DNA targets *in* vivo, then purified from *B. subtilis* and analysed by ChIP-on-chip (34). Under the physiological conditions tested, on the entire *B. subtilis* genome, the only sequence bound by FatR was its known operator site, upstream of the *fatR-cyp102A3* operon (Figure 4A), featuring FatR as a very specific regulator. The binding peak is situated about 200 bp away from the palindromic sequence previously identified as the FatR target (19). The same profile was observed also in the Δ*ptkA* background, showing that when the cells are grown in LB, phosphorylation-based FatR regulation is probably not solicited. We concluded that *fatR-cyp102A3* operon is the main target of FatR regulation, and by consequence, we decided to examine the influence of FatR phosphorylation on *fatR-cyp102A3* expression *in vivo*. For this, we inserted the reporter β-galactosidase gene in the *cyp102A3* locus, in several different backgrounds: wild type, Δ*ptkA, fatR*-Y45F and *fatR*-Y45E. Because FatR operon is known to be derepressed by linoleic acid (16), and also by high salt concentrations (36), we examined the expression from the *fatR-cyp102A3* promoter in uninduced and induced conditions, the latter being in the presence of linoleate and high concentration of NaCl (Figure 4B). All strains exhibited exactly the same growth curve in all conditions, indicating that their growth was not affected (data not shown). In the presence of linoleate and salt, the *fatR-cyp102A3* operon was induced about 2-fold, which is consistent with the very tight repression by FatR observed in the global transcriptome study of *B. subtilis* (36). In the Δ*ptkA* strain, where FatR cannot be inactivated by phosphorylation, the operon was more tightly repressed. It exhibited some induction by fatty acids and salt, but reaching a slightly lower expression level than the wild type. Finally, the strains *fatR*-Y45E and *fatR*-Y45F (which are expected to have impaired DNA binding) exhibited expression levels similar to fully induced wild type already in the absence of linoleate and NaCl. In the presence of linoleate and NaCl, these strains exhibited a strong induction, 3- to 4-fold stronger than in the wild type. The *fatR*-Y45F mutation, lacking the tyrosine 45 hydroxyl group, already provoked a strong derepression of the target operon, and in the mutant *fatR*-Y45E (mimicking also the negative charge of the phosphate) derepression was even stronger. By comparison, deletion of the *fatR* gene led to equally strong derepression in both inducing and non-inducing conditions. These results are consistent with the notion that phosphorylated FatR loses its capacity to repress the *fatR-cyp102A3* operon *in vivo*, and the transcription is activated via PtkA-dependent phosphorylation of FatR.

## DISCUSSION

BY-kinases have been shown to phosphorylate and regulate the activity of many classes of proteins in bacteria (1,2), but to the best of our knowledge, this is the first report showing that tyrosine phosphorylation of a bacterial gene regulator can directly control its DNA-binding properties. PtkA is obviously a promiscuous kinase, involved in modulating the activity of a number of cellular processes. Accordingly, inactivation of this kinase leads to a pleiotropic phenotype (12). A major challenge remains to identify conditions in which this kinase activity is directed towards a specific substrate, and compile the exhaustive lists of its physiological substrates. Reliable experimental approaches for identifying new BY-kinase substrates are few and far in between. In some cases, yeast two-hybrid has been successfully used to detect interaction of bacterial protein kinases with substrates (43). Such interactions are supposed to be short-lived, and two-hybrid cannot be expected to detect them in all cases. For BY-kinases from Firmicutes, the kinase-substrate interaction is in fact a ternary complex, involving also a kinase modulator protein (7). Interestingly, in our two-hybrid assay, the FatR protein interacted with the modulator TkmA (Figure 1A), but not with the kinase PtkA directly. This may suggest a mechanism by which the modulator ‘presents’ this particular substrate to the kinase. *In vitro*, in the presence of TkmA, PtkA was able to phosphorylate FatR (Figure 1B). The site of phosphorylation has been pin-pointed to tyrosine 45 by mass spectrometry (Figure 1C), and assays with point-mutated tyrosine 45 revealed that this is also the major phosphorylation site (Figure 3B). Structural comparisons with FatR homologues indicate that tyrosine 45 is a conserved residue, directly involved in binding DNA via a hydrogen bond (Figure 2). Phosphorylation of key residues at the protein–DNA binding surface is a well-known regulatory mechanism to disrupt interaction with DNA. Probably the most famous case is the MyoD, a nuclear protein from muscle cells, which is phosphorylated in the DNA-binding helix-loop-helix domain, to disrupt interaction with its target sequence E-box (44). Similar regulatory systems exist also in bacteria, for example, *Mycobacterium tuberculosis* DNA-binding protein Rv2175c is phosphorylated on a key threonine residue by the serine/threonine kinase PknL, and this disrupts the interaction of Rv2175c with DNA (45). In this study, we show that the phosphorylation of FatR on tyrosine 45 disrupts its DNA binding, because it ‘masks’ the hydroxyl group involved in a specific hydrogen bond, and introduces electrostatic repulsion with DNA. These two effects can be separated using specific point mutations. Replacement of FatR tyrosine 45 with phenylalanine mimicks just the first effect: the loss of hydroxyl group. As shown in Figure 3C, the lack of hydroxyl group already disrupts DNA binding quite dramatically. The mutant FatR Y45E unites both effects: the loss of hydroxyl group and the addition of a negative charge, thus mimicking the full effect of phosphorylation. To validate this hypothesis, it is crucial to reproduce the regulatory effects *in vivo*. For this, we used the point-mutants *fatR* Y45F and Y45E at the *fatR* locus. To make sure that point-mutations did not disrupt the stability of FatR, ideally one would perform a Western blot analysis in crude extracts, but unfortunately FatR antibodies are not available. Therefore, we performed a CD assay, establishing clearly that there is no loss of secondary structure in FatR Y45F and Y45E. The expression of the FatR target operon was induced about 2-fold in the presence of linoleate and salt, as expected from previous studies (36). In the absence of the kinase PtkA, the operons could still be induced by fatty acids and salt, albeit to a lesser extent than the wild type (Figure 4B). This indicated that PtkA-dependent phosphorylation plays a contributory effect, in parallel with the effect of linoleate and salt. Expression of the target operon in strains *fatR* Y45F and Y45E was strongly induced already in the absence of linoleate and NaCl. However, the effect of fatty acid stress and phosphorylation remained additive, as the addition of linoleate and salt (the ‘induced’ condition) provoked further increase in the expression of *fatR Y*45F and Y45E. The effect of the glutamate mutant was more pronounced in derepressing the *fatR-cyp102A3* operon, which is consistent with its negative charge creating an additional electrostatic repulsion with the DNA. Based on these data, we conclude that PtkA phosphorylates the repressor FatR, and thus contributes to activating the expression from the *fatR-cyp102A3* operon *in vivo*, in stress conditions. Phosphorylation of FatR is not sufficient to completely derepress the operon, as further induction is observed when fatty acids and NaCl are added to strains *fatR* Y45F and Y45E.

Because the residue tyrosine 45 is conserved in its homologues, and that PtkA was able to phosphorylate a number of FatR orthologues from other bacteria (Figure 2D), we propose that this could be a general mechanism for modulating the activity of fatty-acid-displaced regulators in bacteria. We have also shown that a PtkA homologue from *B. megaterium* can phosphorylate its cognate FatR (Bm3R1). In *B. megaterium*, the role of the cytochrome P450 fatty acid monooxygenase CYP102 is to detoxify fatty acids (13,14). The role of FatR-regulated monooxygenase Cyp102A3 in *B. subtilis* is still not clear (16), and other metabolic functions are conceivable. The fact that this operon is also induced by high salt concentrations (36) may suggest a role in a more general stress response. The expression patterns of *fatR-cyp102A3* and *tkmA-ptkA* operons do not correlate in general (36), but they do converge in several conditions, such as heat shock, ethanol stress, hydrogen peroxide stress and spore germination. These conditions may provide leads to identify PtkA activating signals specific for its activity towards FatR.

## SUPPLEMENTARY DATA

Supplementary Data are available at NAR Online.

## FUNDING

Agence Nationale de la Recherche [2010-BLAN-1303-01 to I.M., S.N. and M.F.N.G.]. Funding for open access charge: Institut National de la Recherche Agronomique.

*Conflict of interest statement*. None declared.

## Supplementary Material

Supplementary Data
