# Supplementary material for: Interaction of bacterial fatty-acid-displaced regulators with DNA is interrupted by tyrosine phosphorylation in the helix-turn-helix domain
Source: Nucleic Acids Res. 2013 Aug 10;41(20):9371–81. doi: 10.1093/nar/gkt709 (PMC3814354; doi:10.1093/nar/gkt709)
Supplement: Supplementary Data [file supp_41_20_9371__index.html]

Interaction of bacterial fatty-acid-displaced regulators with DNA is interrupted by tyrosine phosphorylation in the helix-turn-helix domain — Interaction of bacterial fatty-acid-displaced regulators with DNA is interrupted by tyrosine phosphorylation in the helix-turn-helix domain — Supplementary Data 

# Interaction of bacterial fatty-acid-displaced regulators with DNA is interrupted by tyrosine phosphorylation in the helix-turn-helix domain

## Supplementary Data

files

**Files in this Data Supplement:**

- Supplementary Data - pdf file
