# Supplementary material for: Interaction of bacterial fatty-acid-displaced regulators with DNA is interrupted by tyrosine phosphorylation in the helix-turn-helix domain
Source: Nucleic Acids Res. 2013 Aug 10;41(20):9371–81. doi: 10.1093/nar/gkt709 (PMC3814354; doi:10.1093/nar/gkt709)
Supplement: Supplementary Data [file supp_gkt709_nar-00985-f-2013-File006.pdf]

Table S1. Primers used in this study. Mutations are highlighted in bold, restriction sites are underlined and homology with the insertion cassette is in italics.

Name	Sequence	Description
**Amplification primers**		
B.subtilis fatR forward	CGCGGATCCATGATATCCGCATCCAGCAGTAAA TACGACATGATTATG	BamHI
B.subtilis fatR reverse	AAAACTGCAGTTATTTTTGTGATGAATGAAGCTT AATGGCGTCC	PstI
B.cereus fatR forward	CGCGGATCCATGGCTAAAAACAAACAAG	BamHI
B.cereus fatR reverse	AAAACTGCAGTTAAATGATTCTAATAGCG	PstI
B. thuringiensis fatR forward	CGCGGATCCATGGCTAAAAACAAACAAG	BamHI
B. thuringiensis fatR reverse	AAAACTGCAGTTAAATGATTCTAATAGCG	PstI
B. megaterium bm3R1 forward	CGCGGATCCATGGAATCTACTCCAAC	BamHI
B. megaterium bm3R1 reverse	AAAACTGCAGTTAAGATTGTCTGCTAAGTGCTG	PstI
L.casei fatR forward	CGCGGATCCATGAAACCCATCGCCACCCC	BamHI
L.casei fatR reverse	AAAACTGCAGTTATTGAAAGATACTCCTTTC	PstI
B. subtilis yhgD forward	CGCGGATCCATGTCAATAGATCGAAAAAAGC	BamHI
B. subtilis yhgD reverse	AAAACTGCAGTTAGTTCGCTGACAATCCTTTGAC	PstI
B. megaterium bmQ-1130 forward	CGCGGATCCATGGCTCGTAAAAAGAAAG	BamHI
B. megaterium bmQ-1130 reverse	AAAACTGCAGTTAATTACTTCCATAATAATAG	PstI
**Mutagenic primers**		
fatRY45F forward	CGATC**TTT**CGTTATTTTGACAGCAAAG	NA
fatRY45F reverse	TAACG**AAA**GATCGTTCCTGTCCCTACATG	NA
fatRY45E forward	CGATC**GAG**CGTTATTTTGACAGCAAAG	NA
fatRY45E reverse	TAACG**CTC**GATCGTTCCTGTCCCTACATG	NA
**Primers for the DNA probe in gel shift essay**		
BsOP1	TACGGAATGAATATTCATTCCTTT	NA
BsOP2	AAAGGAATGAATATTCATTCCGTA	NA
**Primers for insertion in pMUTIN-2**		
yrhJ forward	CCGGAATTCATGGTTCTCGGTCTTGTATTAAAG	EcoRI
yrhJ reverse	CGCGGATCCTTACGGGGAATCCGCTGATACGT	BamHI
**Primers for *in vivo* mutations**		
fatR in vivo forward	GAGCATATGATGATTGACTCTATA	NA
fatR in vivo reverse	CGCCTCTTTTAAGGCACGGAG	NA
fatRY45E in vivo forward	*GCTCGAATTCACTGGCCGT*CGATC**GAA**CGTTATTT TGACAGCAAAG	NA
fatRY45E in vivo reverse	*CGACCTGCAGGCATGCAAG*CTTTGCTGTCAAAAT AACG**TTC**GATCGTTCCTGTCCCTA	NA
fatRY45F in vivo forward	*GAGCTCGAATTCACTGGCCGTCG*ATC**TTT**CGTTAT TTTGACAGCAAAG	NA
fatRY45F in vivo reverse	*CGACCTGCAGGCATGCAAGCT*TTGCTGTCAAAAT AACG**AAA**GATCGTTCCTGTCCCTA	NA
Δ*fatR in vivo* forward Del-fatR-F	*GAGCTCGAATTCACTGGCCGTCG*CTACCACTATTC CTATGATAGCTCTCGTTCAAACAGGTGATATAG AGATGG	NA
Δ *fatR in vivo* reverse Del-*fatR-R*	*CGACCTGCAGGCATGCAAGCT*ATCATAGGAATAG TGGTAG	NA
yrhH forward	AGGCAGGTGATGGCATGATGAACG	NA
yrhJ reverse	TGAAGGATCCTCAAACCGTTCCG	NA

Table S2. List of *B. subtilis* strains used in this study.

**Strain**	**Description**	**Reference**
Wild type	*B. subtilis 168* trp^+ P_r*::Neo*^{R}	(23)
Δ*ptkA*	Δ*ptkA*	(10)
fatR Y45E	*fatR Y45E* ; constructed as described (23)	This work
fatR Y45F	*fatR Y45F* ; constructed as described (23)	This work
Δ*fatR*	Δ*fatR* ; constructed as described (23)	This work
cypE-lacZ	*cypE::pMUTIN2*	This work
Δ*ptkA cypE-lacZ*	Δ*ptkA cypE::pMUTIN2*	This work
fatR Y45E *cypE-lacZ*	*fatR Y45E cypE::pMUTIN2*	This work
fatR Y45F *cypE-lacZ*	*fatR Y45F cypE::pMUTIN2*	This work
Δ*fatR cypE-lacZ*	Δ*fatR cypE::pMUTIN2*	This work
FatR-SPA	*fatR::pMUTIN2 SPA*	This work
Δ*ptkA* FatR-SPA	Δ*ptkA fatR::pMUTIN2 SPA*	This work
